# Angioleiomyoma of the Auricle: An Unusual Tumor on a Rare Location

**DOI:** 10.1155/2017/8289710

**Published:** 2017-12-10

**Authors:** Jean Kanitakis

**Affiliations:** Department of Dermatology and Dermatopathology, Edouard Herriot Hospital, 69437 Lyon Cedex 03, France

## Abstract

Cutaneous angioleiomyomas (ALMs) (also known as vascular leiomyomas or angiomyomas) are unusual benign tumors of the skin deriving from the muscle layer of dermal blood vessels. They usually manifest as tender subcutaneous nodules, mostly encountered on the legs of adult women in their fifth or sixth life decade. ALMs rarely develop on the head/neck area, and even more rarely (<3% of all cases) on the auricle. Head/neck (including ear) ALMs differ from their more usual leg counterparts in that they are usually painless and do not show a female predominance. The diagnosis is clinically difficult, and most cases are diagnosed by histopathologic examination. A new case of an auricular ALM in a 40-year-old Caucasian man is reported herein, and a brief literature review on this unusual tumor is presented.

## 1. Introduction

Cutaneous angioleiomyomas (ALMs) are a subset of leiomyomas, that is, benign tumors originating from smooth muscle cells present in the skin. ALMs (also known as vascular leiomyomas or angiomyomas) derive from the muscle layer of dermal blood vessels. They manifest typically as solitary, deep dermal, or subcutaneous flesh-colored, well-circumscribed nodules usually located on the lower legs of adult women [1]. They may be recognized clinically because they are usually tender/painful to pressure or even spontaneously, especially when they develop on the lower limbs, but are usually misdiagnosed clinically as epidermoid cysts. In less than 10% of cases, ALMs may develop on the head/neck area, and in that case they are usually painless, rendering the clinical diagnosis even more difficult [2, 3]. Head/neck ALMs have been reported on the nose [4], the cheek [5], the mouth [6], the nasal cavity, the hard palate, the upper lip, the upper eyelid [3], the internal [7] or external [8] auditory canal, and the ear [3, 9–22]. A new case of an immunohistochemically confirmed ALM located on the auricle is presented herein, along with a brief review of the relevant literature.

## 2. Case Presentation

**Figure 1:**
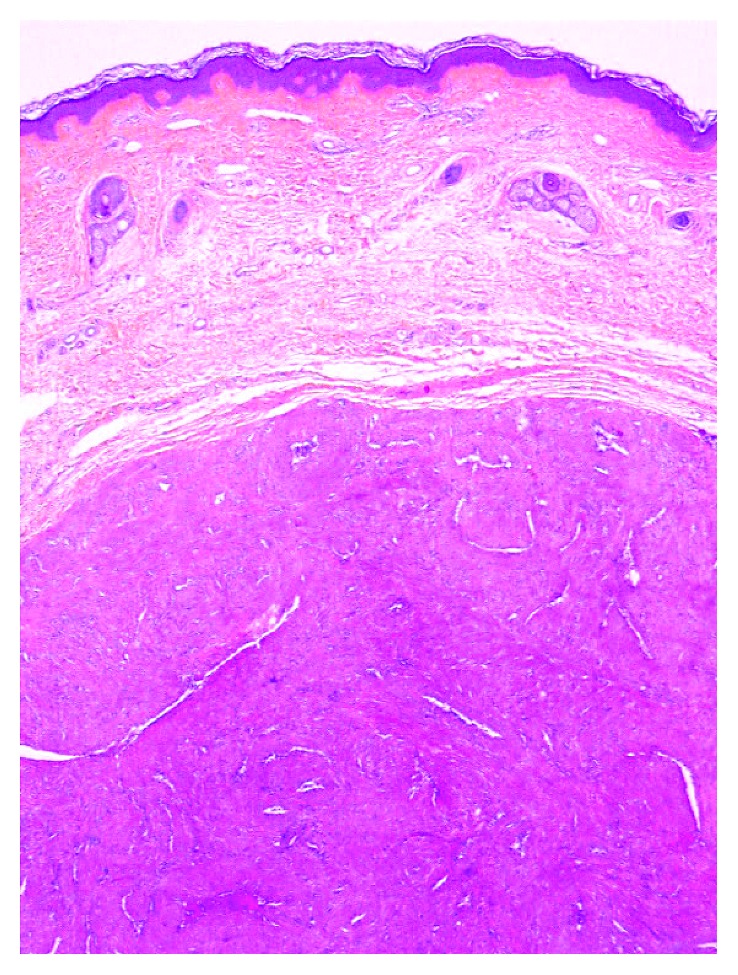
A well-demarcated nodule is seen in the dermis (original magnification: ×40).

**Figure 2:**
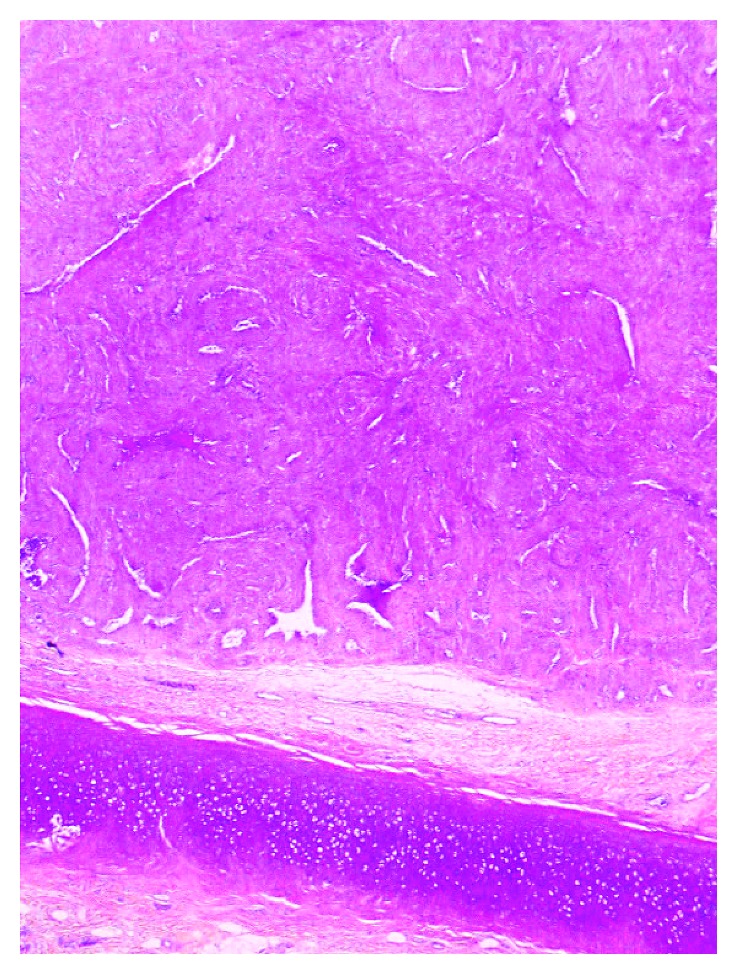
The dermal tumor spares the underlying ear cartilage (original magnification: ×40).

**Figure 3:**
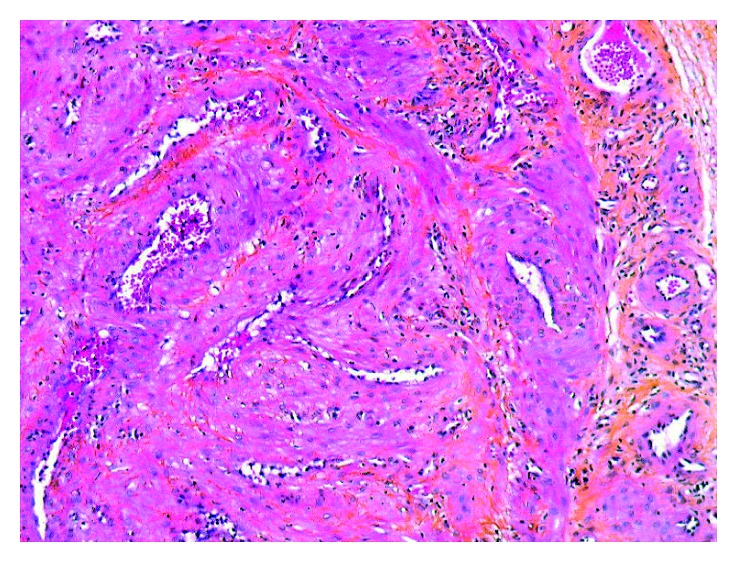
At higher magnification, the tumor consists of spindle cells with eosinophilic cytoplasm and blunt nuclei, forming fascicles surrounding the vessels with thick muscular walls (hematoxylin-eosin-saffron stain, original magnification: ×250).

**Figure 4:**
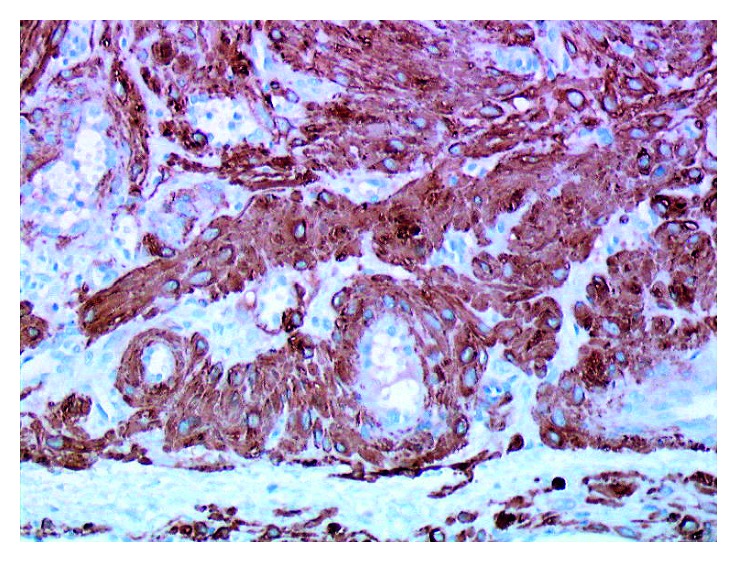
By immunohistochemistry, tumor cells express caldesmon (immunoperoxidase revealed with aminoethylcarbazole) (original magnification: ×250).

A 40-year-old man of North African descent noted the progressive development, since one year, of a nodule on the rim of the helix of his right ear. The lesion had developed spontaneously and was asymptomatic, but its size was somewhat fluctuating according to the patient. Physical examination showed a ca. 1 cm firm nodule of the posterior aspect of the ear with a slightly bluish hue. The patient was otherwise in good condition, and his family history was unremarkable. The lesion was excised under local anesthesia and submitted for pathological examination, with the probable clinical diagnoses of follicular cyst or angioma. Microscopic examination showed a roundish, well-demarcated nodular tumor invading the mid- and deep-dermis, sparing the underlying ear cartilage (Figures 1 and 2). It was made of elongated cells with an eosinophilic cytoplasm and centrally located, blunt nuclei with rounded, cigar-like contours. The cells formed interlacing bundles assuming often a concentric or whorled appearance. Several vascular channels were seen within the nodule, and at the periphery of the lesion, several small round vessels with a thick smooth-muscle wall were present, often merging with the main spindle-cell population of the nodule (Figure 3). The immunohistochemical study showed that the tumor cells expressed smooth-muscle markers, that is, caldesmon (Figure 4), desmin, and smooth-muscle actin, but not keratin. These findings were diagnostic of ALMs (solid type). The patient was lost to further follow-up.

## 3. Discussion

Cutaneous ALMs are uncommon tumors. A review of the files of our dermatopathology laboratory revealed 36 cases diagnosed during 16 years (1999–2015). In this series, the mean age of patients was 59 years (range, 36–79), and there was a clear female predominance (77.8%). The location of ALMs (mentioned in 35/36 cases) was on the lower limbs (57%), including the leg/shin (34%), the ankle/heel/foot (23%), the knee (14%), and the thigh (2.85%). The remaining cases were located on the forearm and elbow (23%), the palm (2.85%), and the ears (2 cases, i.e., 5.8%—the second of these cases was located on the earlobe of a 58-year-old woman and had been submitted to our laboratory by another institution; therefore, no other clinical data were available). These data are in agreement with those of the literature, namely, a large study of 562 cases [2] that reported ALM location on the lower limbs in 67% of cases, the upper limbs in 22%, the head/neck region in 8.5%, the trunk in 2.5%, and the ear in 2.8% of cases.

The clinical appearance of ALMs of the ears is not very specific. The lesion manifests in adults as a single nodular growth with occasionally a bluish red hue that may fluctuate in size, as in our patient. Auricular ALMs may be located on the helix [12, 15], the antihelix [16, 18], the lobule [11, 13], or the external auditory canal [8]. Most cases appear during adulthood, typically during the fifth or sixth life decade, as in our patient, but one case was reportedly present since childhood [11]. Although rare cases of painful ALMs on the ears have been reported [18], head/neck ALMs are usually painless [3, 12, 13, 15–17, 19, 22], contrasting with ALMs of the limbs, which are usually tender or painful upon pressure. ALMs of the external auditory canal can cause hearing loss [8]. Furthermore, contrasting with ALMs of the lower limbs [3], ALMs of the head/neck (including the ear) show a male predominance [3, 11–13, 15, 19, 22]. Clinically, ALMs must be differentiated from other benign tumors of the auricle, namely, hemangiomas, glomus tumors, epidermoid cysts, auricular pseudocysts, angiolymphoid hyperplasia with eosinophilia, and neuro(fibro)mas. The diagnosis is generally made by histological examination. The pathological aspect includes a well-demarcated dermal tumor consisting of smooth muscle cells, in-between which a variable amount of vascular channels is seen. Three subtypes have been reported, including a solid type (smooth muscle bundles surround numerous small slit-like vessels), a cavernous type (dilated vessels with a thick muscular wall merging with the intervascular smooth muscle cells), and a venous type (thick-walled vessels easily distinguished from the intervascular smooth muscles) [1]. The commonest type is the solid one, followed by the venous and the cavernous type. Rarer variants include the epithelioid and the pleomorphic type [1]. The pathological diagnosis of ALMs is usually easy, although the lesions may be mimicked by other spindle-cell proliferations (such as dermato(myo)fibromas, glomus tumors, myopericytomas, hemangiomas, or keloids), and as a matter of fact, some cases published with the diagnosis of leiomyomas seem to correspond pathologically to keloids [23]. Immunohistochemistry is in such doubtful cases helpful in establishing the diagnosis, showing tumor cells to express at least one of the muscle-cell markers, that is, actin, desmin, calponin, and caldesmon. The course of ALMs of the ear is usually uneventful as simple surgical excision is curative, the rate of recurrences of ALMs in general being less than 0.4% [2].

Although ALMs only rarely develop on the ear, they should be included in the clinical differential diagnosis of tumors developing in this anatomic zone. The definite diagnosis requires histopathological examination of a biopsy (or excision) specimen.
